# Pan-genome Analysis of Ancient and Modern *Salmonella enterica* Demonstrates Genomic Stability of the Invasive Para C Lineage for Millennia

**DOI:** 10.1016/j.cub.2018.05.058

**Published:** 2018-08-06

**Authors:** Zhemin Zhou, Inge Lundstrøm, Alicia Tran-Dien, Sebastián Duchêne, Nabil-Fareed Alikhan, Martin J. Sergeant, Gemma Langridge, Anna K. Fotakis, Satheesh Nair, Hans K. Stenøien, Stian S. Hamre, Sherwood Casjens, Axel Christophersen, Christopher Quince, Nicholas R. Thomson, François-Xavier Weill, Simon Y.W. Ho, M. Thomas P. Gilbert, Mark Achtman

**Affiliations:** 1 Warwick Medical School, University of Warwick, Gibbet Hill Road, Coventry CV4 7AL, UK; 2 Centre for GeoGenetics, Natural History Museum of Denmark, University of Copenhagen, Øster Voldgade 5-7, 1350 Copenhagen, Denmark; 3 Unité des Bactéries Pathogènes Entériques, Institut Pasteur, Paris, France; 4 Department of Biochemistry and Molecular Biology, University of Melbourne, Parkville, Victoria 3010, Australia; 5 Wellcome Trust Sanger Institute, Cambridge, UK; 6 NTNU University Museum, N-7491 Trondheim, Norway; 7 Department of Archaeology, History, Cultural Studies and Religion, University of Bergen, Post Box 7805, 5020 Bergen, Norway; 8 Pathology Department, University of Utah School of Medicine, Salt Lake City, UT 84112, USA; 9 School of Life and Environmental Sciences; University of Sydney, Sydney NSW 2006, Australia

**Keywords:** Ancient DNA, paratyphoid fever, enteric fever, Salmonella enterica, genomic stability, population genomics, pan-genome, historical infections, tMRCA of bacterial pathogens, host jump

## Abstract

*Salmonella enterica* serovar Paratyphi C causes enteric (paratyphoid) fever in humans. Its presentation can range from asymptomatic infections of the blood stream to gastrointestinal or urinary tract infection or even a fatal septicemia [1]. Paratyphi C is very rare in Europe and North America except for occasional travelers from South and East Asia or Africa, where the disease is more common [2, 3]. However, early 20^th^-century observations in Eastern Europe [3, 4] suggest that Paratyphi C enteric fever may once have had a wide-ranging impact on human societies. Here, we describe a draft Paratyphi C genome (Ragna) recovered from the 800-year-old skeleton (SK152) of a young woman in Trondheim, Norway. Paratyphi C sequences were recovered from her teeth and bones, suggesting that she died of enteric fever and demonstrating that these bacteria have long caused invasive salmonellosis in Europeans. Comparative analyses against modern *Salmonella* genome sequences revealed that Paratyphi C is a clade within the Para C lineage, which also includes serovars Choleraesuis, Typhisuis, and Lomita. Although Paratyphi C only infects humans, Choleraesuis causes septicemia in pigs and boar [5] (and occasionally humans), and Typhisuis causes epidemic swine salmonellosis (chronic paratyphoid) in domestic pigs [2, 3]. These different host specificities likely evolved in Europe over the last ∼4,000 years since the time of their most recent common ancestor (tMRCA) and are possibly associated with the differential acquisitions of two genomic islands, SPI-6 and SPI-7. The tMRCAs of these bacterial clades coincide with the timing of pig domestication in Europe [6].

## Highlights

• *Salmonella enterica* aDNA sequences were found within 800-year-old teeth and bone
• The invasive Para C lineage was defined from 50,000 modern *S. enterica* genomes
• The Para C lineage includes Ragna, the aDNA genome, and human and swine pathogens
• Only few genomic changes occurred in the Para C lineage over its 3,000-year history

## Teaser

Zhou et al. reshape our understandings of the origins of an invasive bacterial pathogen, *Salmonella enterica* Paratyphi C, by combining a reconstructed pan-genome from an 800-year-old skeleton in Norway with 221 genomes from modern bacteria.

## Results and Discussion

**Figure 1:**
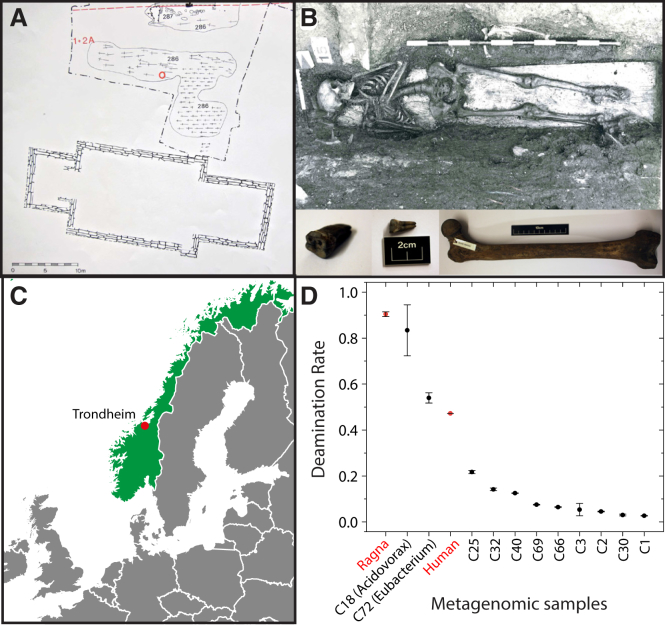
Geographic, Archaeological, and Metagenomic Features of Skeleton SK152 (A) Excavation site (Folkebibilotekstomten, 1973–1985) of the church cemetery of St. Olav in Trondheim, Norway. The burial location of SK152 (red circle) belongs to a building phase that has been dated archaeologically [11] to 1200 CE (range 1175–1225). (B) Entire skeleton (top) and femoral long bone plus two teeth from which *Salmonella* DNA was extracted (bottom). (C) Map of Europe surrounding Norway (green) and location of Trondheim (red). (D) Deamination rate for metagenomic reads in the *Salmonella* Paratyphi C Ragna genome, human DNA and 11 single genome assemblies (Cxx) identified by Concoct [12]. C18 (*Acidovorax*) and C72 (*Eubacterium*) show high levels of deamination rates, as do reads from humans or Ragna, while the other assemblies have low levels and likely represent modern environmental bacteria. Data: Mean plus error bars showing standard deviations. See also Figure S2 for properties of aDNA reads.

According to historical records [7], humans have long been afflicted by bacterial infections, yet genomic analyses of extant bacterial pathogens routinely estimate a tMRCA of no more than a few centuries [8]. In general, evolutionary trees contain a stem group, which may include lineages that are now rare or extinct, as well as the crown group of extant organisms. Historical reconstructions based only on the crown group ignore the older sub-lineages in the stem group and thereby provide an incomplete picture of the older evolutionary history of the pathogen. In contrast, analyses of ancient DNA (aDNA) can shed light on additional millennia of bacterial pathogen evolution that occurred prior to the origin of the crown group [9, 10]. We therefore searched for ancient bacterial lineages by scanning metagenomic sequences from teeth and long bones of 33 skeletons who were buried between 1100 and 1670 CE in Trondheim, Norway [11] (Figures 1A and 1C).

SK152 is the skeleton of a 19- to 24-year-old woman of 154 ± 3 cm height who was buried in 1200 ± 50 CE, according to archaeological investigations [11]. Calibrated radiocarbon (^14^C) dating of two teeth estimated her burial as 100–200 years earlier (Figure S1B); this minor discrepancy may reflect the reservoir effect on radiocarbon dating from a predominant diet of fish products [13]. Based on δ^18^O_carbon_ isotopic measurements from her first and third molars, this woman likely migrated from the northernmost inland areas of Scandinavia or Northwest Russia during her childhood and arrived in Trondheim by her early teens [14] (Figure S1A). The skeleton rested on a wooden plank (a symbolic coffin) in a grave filled and covered with anoxic, acidic, and waterlogged wood chips and soil (Figure 1B).

### Metagenomic Reads Associated with SK152

We identified 266 *Salmonella enterica* sequence reads in one tooth from skeleton SK152 and many more *Salmonella* sequences from additional libraries from SK152 teeth and bone, but not from dental calculus (Table 1). We attempted to reconstruct this *Salmonella* genome (henceforth designated “Ragna”) from the SK152 sequence libraries. Concoct [12] was able to reconstitute 11 near-complete microbial genomes from those data (Figure 1D). Nine of these genomes likely reflect recent soil contamination because their 5′-single-stranded deamination rates were low [15], with DNA damage < 0.22, versus values of 0.47 for human DNA and 0.9 for Ragna.

**Table 1:** Reads Specific to *S. enterica* within Metagenomic Sequences of Samples from SK152

					Reads specific for *S. enterica* (Ragna)				
**Source**	**No. of libraries**	**Total unique reads**	**Total human reads**	**Total non-human reads**	**% of all reads**	**% duplicates**	**No. of unique reads**	**Mean read length (bp)**	**Genome coverage**

Upper 3^rd^ left molar root and pulp	2	237,735,419	58,068,866	179,666,553	0.050	77	26,853	56	0.29
Upper 2^nd^ right molar dentine/cementum	4	1,077,156,946	127,986,372	949,170,574	0.183	53	920,267	35	6.39
Upper 2^nd^ right molar pulp	1	119,308,674	26,725,529	92,583,145	0.088	26	77,928	43	0.60
Femoral long bone	1	73,372,819	12,765,129	60,607,690	0.013	49	5,060	41	0.04
Dental calculus (multiple teeth)	1	235,375,745	20,737,655	214,638,090	0.000	N/A	N/A	N/A	N/A
Total:	9	1,742,949,603	246,283,551	1,496,666,052	0.126	53	1,030,108	36	7.32

See also Figure S1 for archaeological information and dating estimates for the burial of SK152. N/A: Not applicable

The two other assembled genomes exhibited high levels of DNA damage and are thus likely to have been endogenous to this corpse since burial. Genome C72, from a novel species of *Eubacterium*, was found almost exclusively in dental calculus, and these bacteria may have been a component of a biofilm associated with periodontal disease, as are other *Eubacterium* species [16]. Genome C18 belongs to *Acidovorax*, which is associated with plant pathogens [17] and may have been introduced with the wood chips that covered the skeleton. We therefore decided to reconstruct the Ragna genome by read mapping against its close relatives within *S. enterica*.

### The Para C Lineage

**Figure 2:**
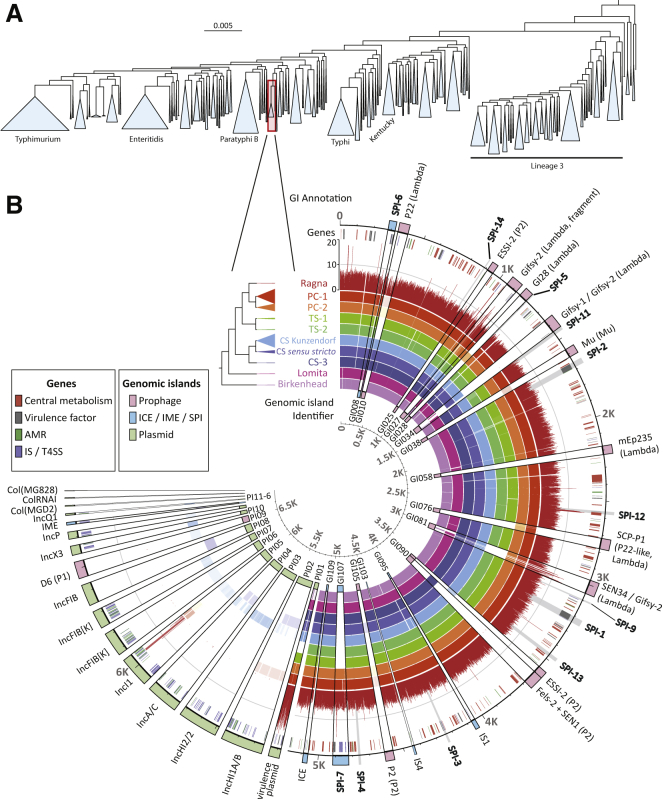
Genomic Phylogenies of *Salmonella enterica* and the Para C Lineage (A) Maximum-likelihood phylogeny of 2,964 representative genomes of *S. enterica* subspecies *enterica*. Each genome is a representative of one ribosomal multi-locus sequence typing (rMLST) sequence type. Blue triangles indicate common lineages containing numerous genomes, including the Para C lineage. Several lineages are associated with common serovars, as indicated by horizontal labels. Red rectangle: Para C lineage plus the outgroup Birkenhead. (B) Pan-genomic contents for 6,665 pan-genes in 222 genomes of the Para C lineage, including Ragna, plus Birkenhead, with one stroke per gene. Circles (inner to outer); Circle 1: sixteen major, variably present chromosomal genomic islands (GI008–GI109) followed by sixteen cytoplasmic plasmids, circular phages plus one IME (PI01–PI16), color-coded as in the genomic islands key. Circles 2–10: the frequency of presence or absence of each gene per sub-lineage within the phylogram is indicated by color opacity. Circle 11: coverage of aDNA reads per gene within Ragna (scale 0–20 reads at 12:00). Circle 12: genes color-coded as in the genes key. Circle 13: traditional designations for GIs, PIs, and other variably present genomic elements (Table S3). Gray wedges: SPIs. See also Table S1 for summary statistics on the sources and dates of collection of the bacterial strains and Table S5 for the metadata for each individual genome.

Identifying close relatives of the Ragna genome required an overview of the genetic diversity of *S. enterica* subspecies *enterica.* To this end, we inferred phylogenetic trees of 2,964 genomes that represented the diversity of 50,000 strains of *S. enterica* in EnteroBase [18]. These contained 711,009 single-nucleotide polymorphisms (SNPs) in 3,002 core genes (2.8 Mb). A maximum-likelihood tree of the concatenated core genes revealed the existence of multiple, discrete lineages (Figure 2A). The initial sequence reads from Ragna were most closely related to one of these lineages, the “Para C lineage.” The Para C lineage is comprised of monophyletic clades of serovars Paratyphi C, Choleraesuis, and Typhisuis, which were already known to be related by lower resolution analyses [3], plus one genome of the extremely rare serovar Lomita (Figure 2). Paratyphi C only infects humans, but serovar Choleraesuis is associated with septicemia in swine (and occasionally humans) and Typhisuis is associated with epidemic swine salmonellosis (chronic paratyphoid) in domestic pigs [2, 3]. Although these other serovars continue to cause disease in southern and eastern Asia, Choleraesuis is rare in Europe today except in wild boar [5], and Typhisuis has been eradicated from European pigs. For our further phylogenetic analyses, we included two genomes of serovar Birkenhead as an outgroup because they were the closest genetic relatives of the Para C lineage (Figure 2).

Reliable inference of the evolutionary timescale and phylogeographic history of the Para C lineage depends on a broad temporal and spatial range of sources for the bacterial strains. However, EnteroBase only contained 100 *Salmonella* genomes from the Para C lineage, and they were of limited geographical and temporal diversity. We therefore combed the strain collection at the Institut Pasteur, Paris, and sequenced 119 additional Para-C-lineage genomes from diverse, historical sources. Our final dataset comprised 219 modern Para-C-lineage genomes, isolated between 1914 and 2015 from multiple continents (Table S1).

A maximum-likelihood tree of the core SNPs within the 219 genomes showed that they fell into well-defined sub-lineages within each serovar (Paratyphi C: PC-1, PC-2; Choleraesuis: CS Kunzendorf, CS *sensu stricto*, CS-3; Typhisuis: TS-1, TS-2) (Figure 2B). We also calculated a pan-genome, which was used to map the SK152 metagenomic reads after initial processing and de-duplication. Mapping identified 1,030,108 unique *Salmonella* reads in teeth (0.05%–0.18% of all reads) or the femur (0.01%), but not in dental calculus (Table 1). All of these reads were specific to Paratyphi C and covered 98.4% of a reference Paratyphi C genome (RKS4594) with a mean read depth of 7.3-fold (Table 1). To avoid spurious SNP calls associated with DNA damage, we only called SNPs in the Ragna genome that were covered by at least two reads, resulting in 95% coverage of RKS4594 (Figure S2).

Our data demonstrate that Paratyphi C bacteria caused human infections in Norway 800 years ago, and their presence in both teeth and bones suggests that SK152 died of septicemia associated with enteric fever. Paratyphi C aDNA from 1,545 CE has also been recently described from mass graves in Mexico [19], consistent with a continuous history of systemic human disease associated with this pathogen.

### Pan-genomic Stability

**Figure 3:**
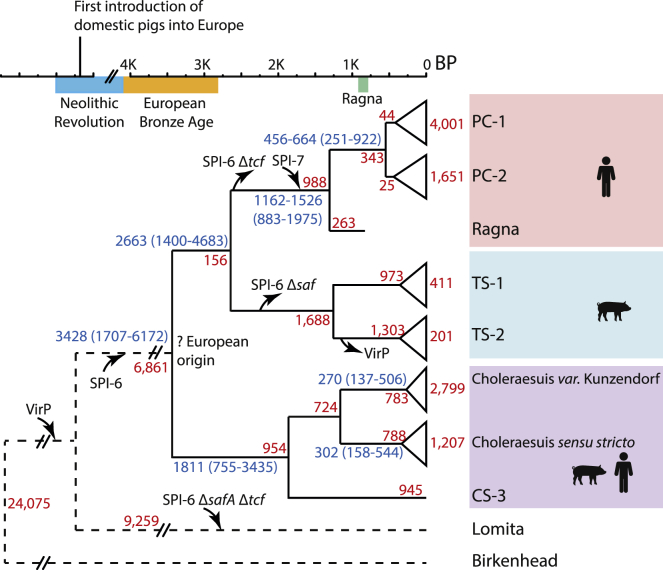
Cartoon of the Evolutionary History of the Para C Lineage on a Time Frame for Human History in Europe BP: before present. The tree is annotated with the acquisitions of the virulence plasmid (VirP), SPI-6 and SPI-7 as annotated by inward arrows, and deletions of parts of SPI-6 as annotated by outward arrows. Numbers in blue indicate date estimates and their 95% credible intervals (parentheses) according to a Bayesian phylogenetic approach. Red numbers indicate numbers of substitution events for non-recombinant core SNPs except for Lomita and Birkenhead, where they indicate total core SNPs. The host specificities of the individual serovars are indicated by cartoons at the right. See also Figure S3 for the timing of individual changes in pseudogenes and gene gain/loss, Figure S4 for the locations of variable genes by lineage within SPI-6, Table S3 for the properties of individual genomic islands, and Table S4 for detailed estimates of tMRCAs. See also Table S2.

The selective pressures associated with local ecological interactions are thought to cause variation of gene content in microbes [20]. We therefore anticipated that 800 years of evolution would have resulted in dramatic differences in gene content between Ragna and modern Paratyphi C genomes, and we expected even greater differences between Paratyphi C and the other clades of the Para C lineage. Surprisingly, 78% of the 4,388 ± 99 genes (total length 4.8 ± 0.08 Mb) in a Para-C-lineage genome were intact core genes, and only 604 core SNPs distinguished Ragna from the MRCA of modern Paratyphi C (Figure 3). Some core genes are universally present in the Para C lineage plus Birkenhead even though they belong to mobile genetic elements that are variably present in other *Salmonella*, e.g., the pathogenicity islands SPI-1 to SPI-6, SPI-9, and SPI-11 to SPI-14 (Figure 2B). Similarly, the virulence plasmid was present throughout the Para C lineage except for Typhisuis sub-lineage TS-2. A further constant feature of the Para C lineage was the absence of genes encoding typhoid toxin, which is thought to trigger enteric fever by serovars Typhi and Paratyphi A [21].

Other studies have indicated that microbial host adaptation is accompanied by the accumulation of pseudogenes [22], driven by the streamlining of genes that are no longer necessary for the infection of multiple hosts [23], or by rewiring of transcriptional regulation [24]. The 2,964 representative *Salmonella* genomes contained a median of 40–60 pseudogenes. The numbers of pseudogenes were unexceptional for the most recent common ancestors (MRCAs) of the Para C lineage (25 pseudogenes) or of Paratyphi C, Choleraesuis plus Typhisuis (69 pseudogenes) (Figure S3C), suggesting that neither of these MRCAs was adapted to any particular host. However, the MRCAs of the individual serovars may well mark the beginnings of host adaptation because they were associated with higher numbers of pseudogenes (Choleraesuis: 95; Paratyphi C: 116; Typhisuis: 181). These pseudogenes may simply represent functions that are not required for infection of their individual hosts or may even have contributed to host specificity.

We also attempted to identify mobile genetic elements in the accessory genome of the Para C lineage that could account for their differential host specificities. The 3,901 accessory genes clustered together within 227 GIs (genomic islands and other mobile elements), including 37 plasmids, 32 prophages, 16 IMEs (integrative and mobilizable elements), SPI-5 to SPI-7, and two ICEs (integrative and conjugative elements) (Table S2). Parts or all of these GIs were acquired or lost on 311 independent occasions. However, at least 60% of gains or losses are unlikely to be important for host specificity because they were restricted to a single genome (Table S2), and most gains or losses were very recent (Figure S3B). Most of the other gains or losses are also unlikely to represent successful evolutionary changes in virulence or host specificity because, as in other *Salmonella* [25, 26, 27], they were restricted to individual sub-lineages, and sister sub-lineages differing in the possession of those genes are also prevalent in invasive disease (Figure 2B). For example, Paratyphi C is more virulent for mice after lysogenization upstream of *pgtE* by a P22-like prophage, SCP-P1 [28] (here GI076), whose gene product prevents opsonization [29]. However, GI076 is absent from half of the Paratyphi C genomes, including Ragna (Figure 2B). Similarly, the *fimH102* allele of a type 1 fimbrial adhesin facilitates specific adhesion to porcine cells by serovar Choleraesuis [30], but *fimH* is totally lacking in CS *sensu stricto*. Indeed, none of the inferred virulence factors and GIs seemed likely to be consistently related to differential virulence or host specificity (Table S3), with the notable exceptions of SPI-7 and SPI-6.

SPI-7 (GI107) is a pathogenicity island which encodes the Vi capsular polysaccharide in serovars Typhi, Paratyphi C (including Ragna), and Dublin. Vi might promote enteric fever in humans because it prevents the opsonization and clearance that is triggered by binding of the C3 component of complement to lipopolysaccharide [31]. The presence of SPI-7 in all Paratyphi C, and its absence from all Typhisuis and Choleraesuis, suggests that SPI-7 was acquired prior to the expansion of serovar Paratyphi C and also suggests an association with its human specificity. A 5-gene deletion within SPI-7 is present throughout sub-lineage PC-1, but this does not affect the production of Vi polysaccharide [3].

SPI-6 (GI008) is present throughout the Para C lineage. It encodes a type-6 secretion system (T6SS), as well as *Salmonella* atypical fimbriae (*saf*) and Typhi colonization factor (*tcf*) (Figure S4). T6SS systems encode intracellular, inverted bacteriophage-tail-like structures that can inject lethal effectors (TaeX) into neighboring eukaryotic and bacterial cells [32]. SPI-6 contributes to the gastrointestinal colonization and pathogenesis of mice [33] and chickens [34] by serovar Typhimurium. Expression of *tcf* resulted in specific adhesion of *Escherichia coli* to human epithelial cells [35], and *saf* mutations reduced gastrointestinal colonization of pigs by serovar Choleraesuis [36]. The *tcf* and *saf* genes are variably present within SPI-6 in the multiple serovars within the Para C lineage (Figure S4) and might therefore also account for their differential host specificity. A parsimonious interpretation of the origins of this diversity is that an intact SPI-6 was initially acquired by horizontal transfer after Lomita branched off, followed by successive, internal deletions prior to the MRCAs of Paratyphi C and Typhisuis. Alternatively, multiple SPI-6 variants might each represent an independent horizontal transfer event.

### Evolutionary Timing

These observations immediately raised the question of evolutionary timescales, including the ages of individual serovars. We confirmed the existence of significant temporal signal according to root-to-tip distances [37] and date randomization tests [38] with the non-recombinant SNPs from the Para-C-lineage genomes from 10 independent samples of Paratyphi C (both including and excluding Ragna) and also from the Para C lineage (without Lomita, which lacks a collection date). We then dated key stages in the evolution of the Para C lineage by a Bayesian phylogenetic approach (Figure 3). The optimal model for Paratyphi C (strict clock) yielded a median substitution rate of 7.9 × 10^−8^ substitutions/site/year (95% credible interval: 5.3 × 10^−8^-1.1 × 10^−7^), slightly lower than the median rate estimated according to the optimal model (relaxed) for the Para C lineage (1.5 × 10^−7^; 95% CI: 6.9 × 10^−8^ – 2.5 × 10^−7^) (Table S4). The age of the crown group encompassing modern isolates of Paratyphi C was dated at 456–664 BP (95% CI: 251–922) and its split from Ragna at 1,162–1,526 (95% CI: 883-1,975) (Table S4). Thus, the addition of the Ragna genome sheds light on about 800 additional years of evolutionary history of Paratyphi C.

Our estimate of the timescale allowed us to investigate the rates of gain or loss events and pseudogene formation within Paratyphi C relative to the accumulation of SNPs (Figure S3). The results showed that pseudogenes accumulated slowly and continuously (3–5 per 100 SNPs) since the tMRCA until the last 200 years, when their rate accelerated to 7 pseudogenes/100 SNPs (Figure S3A). The rate of gene gain or loss was also low (1.3 genes/100 SNPs) until 400 years ago, increased to 15–20 genes/100 SNPs and increased even further to 49 genes/100 SNPs in the last 50 years before laboratory cultivation (Figure S3B). Similar results were obtained for the other sub-lineages within the Para C lineage (Figures S3C and S3D). Recent gene gain or loss has also been noted in *S. enterica* serovars Paratyphi A [25] and Agona [26] and was attributed to frequent acquisitions of selfish DNA, which were also subsequently rapidly lost. Our results confirm that genomic islands are indeed highly variable in modern isolates. However, they clearly show that they were lost or gained much less often in previous millennia, which were marked by relative pan-genomic stability.

The age of the stem lineage of Paratyphi C stretches back to its differentiation from serovar Typhisuis, about 2,663 years (95% CI: 1,440–4,683), during which time ∼1,350 SNPs were accumulated prior to the MRCAs of the PC-1 and PC-2 clades (Figure 3). In turn, the estimated tMRCA for Paratyphi C, Typhisuis plus Choleraesuis was approximately 3,428 years ago (95% CI: 1,707–6,172) and that ancestor evolved in Europe according to three independent Bayesian and maximum-likelihood methods. A European ancestry is consistent with the existence of Paratyphi C and enteric fever in northern Norway 800 years ago and also with enteric fever caused by Paratyphi C bacteria in Mexico in 1,545 CE, which was inferred to have been introduced by Europeans [19]. We also note that serovars Choleraesuis and Typhisuis infect swine and that their tMRCAs overlap with the Neolithic domestication of pigs from wild boar in Europe [39] (Figure 3). It is therefore possible that Paratyphi C represents the host specialization to humans of a zoonotic pathogen of domesticated animals. Alternatively, Typhisuis may have specialized from a generalist life style to a host specificity for swine.

Our identification of the Ragna genome from a young woman who died in 1,200 CE provides insights on the genomic contents of the stem lineage of serovar Paratyphi C. The Ragna genome also demonstrates that salmonellosis was a deadly invasive disease of humans for centuries before its first recognition by physicians. Our analyses show that reconstructing the long-term evolutionary history of bacterial pathogens benefits dramatically from comparisons of metagenomic data from ancient samples with population genetic data from present-day bacteria. The close relationship between clades of the Para C lineage that differ in host specificity triggered intriguing speculations about historical host jumps during the Neolithic period between humans and their domesticated animals. Our results also indicate that both the core and accessory genome of bacterial pathogens can be remarkably stable over millennia and that much of the dramatic variation between extant genomes represents transient genetic fluctuation, whose evolutionary relevance to ecological fitness is uncertain.

## STAR★Methods

### Key Resources Table

**Table:** 

REAGENT or RESOURCE	SOURCE	IDENTIFIER
**Bacterial and Virus Strains**		

Table S5. Bacterial Strains and genomes.xlsx	This paper	N/A

**Deposited Data**		

Ancestral reconstruction 1 Maximum likelihood phylogeny and geographical origins derived from 49,610 non-recombinant, non-repetitive core SNPs within the Para C lineage plus Birkenhead	This paper	http://wrap.warwick.ac.uk/101809
Ancestral reconstruction 2 Geographic inference of the sources of clades within the Para C lineage	This paper	http://wrap.warwick.ac.uk/101810
Concoct 1 Single core gene (SCG) frequencies in the 76 Concoct clusters generated after binning contigs	This paper	http://wrap.warwick.ac.uk/101811
Concoct 2 Statistics after clustering all metagenomes with MEGAHIT	This paper	http://wrap.warwick.ac.uk/101812
Concoct 3 Taxonomic assignments for 11 metagenome assembled genomes (MAGs)	This paper	http://wrap.warwick.ac.uk/101813
Concoct 4 Single-stranded deamination rates for Ragna, human DNA and 11 MAGs	This paper	http://wrap.warwick.ac.uk/101814
Concoct 5 Genomic coverage of 11 MAGs by source and sequencing library	This paper	http://wrap.warwick.ac.uk/101815
Concoct 6 Bacterial taxa found by Kraken in SK152 metagenomes at ≥ 0.02% frequency.	This paper	http://wrap.warwick.ac.uk/101816
Concoct 7 Eleven MAGs Contigs.	This paper	http://wrap.warwick.ac.uk/101817
Concoct 8 Alignments and phylogenies of SCGs across the Tree of Life.	This paper	http://wrap.warwick.ac.uk/101818
Date estimation 1 Date randomization tests for the temporal signal in multiple replicate datasets that were analyzed by Beast	This paper	http://wrap.warwick.ac.uk/101819
Date estimation 2 Clock-like behavior of root to tip distances for Ragna, Tepos-14 and Tepos-35 plus modern genomes from the Para C lineage or Paratyphi C.	This paper	http://wrap.warwick.ac.uk/101820
Date estimation 3 Comparison of MRCA dating estimates by Beast using ancient plus modern, and only modern strains	This paper	http://wrap.warwick.ac.uk/101821
Date estimation 4 Raw Maximum Clade Credibility trees from BEAST inferences.	This paper	http://wrap.warwick.ac.uk/101822
ParaC genome SNPs 1 Numbers of mutational and recombinational SNPs per clade within the Para C lineage plus Birkenhead	This paper	http://wrap.warwick.ac.uk/101831
ParaC genome SNPs 2 SNPs in the core genome of the Para C lineage plus Birkenhead.	This paper	http://wrap.warwick.ac.uk/101832
ParaC genome SNPs 3 Maximum likelihood phylogeny and inferred geographical origins based on 49,610 non-recombinant, non-repetitive core SNPs from the Para C lineage plus Birkenhead.	This paper	http://wrap.warwick.ac.uk/101833
ParaC genome SNPs 4 Venn diagram of substitutions scored as recombinational or mutational by RecHMM, ClonalFrameML and Gubbins.	This paper	http://wrap.warwick.ac.uk/101834
ParaC Pan-genome 1 Gain and loss of genomic islands shown on a maximum-likelihood radial phylogeny derived from 49,610 non-recombinant, non-repetitive core SNPs within the Para C lineage plus Birkenhead	This paper	http://wrap.warwick.ac.uk/101823
ParaC Pan-genome 2 Summary statistics for pan-genomic contents of Ragna plus 219 modern genomes of the Para C lineage	This paper	http://wrap.warwick.ac.uk/101824
ParaC Pan-genome 3 Gene gain/loss events and numbers of pseudogenes by sub-lineage	This paper	http://wrap.warwick.ac.uk/101825
ParaC Pan-genome 4 The pan-genome of the Para C lineage based on 6,665 single copy genes from 220 genomes.	This paper	http://wrap.warwick.ac.uk/101826
ParaC Pan-genome 5 Sequences, original gene names and annotations of the reference sequences for 6,665 pan genes in the Para C lineage.	This paper	http://wrap.warwick.ac.uk/101827
ParaC Pan-genome 6 Pseudogenes associated with sub-lineages and clades of the Para C lineage.	This paper	http://wrap.warwick.ac.uk/101828
ParaC Pan-genome 7 Genomic islands and plasmids.	This paper	http://wrap.warwick.ac.uk/101829
ParaC Pan-genome 8 Data and script for extracting rate of gene gain/loss and pseudogenes from the branches of an ML tree. See readme.txt for instructions.	This paper	http://wrap.warwick.ac.uk/101830
*Salmonella* supertree 1 Metadata for 2,964 genomes that are representative of the genomic diversity of *Salmonella enterica* subsp. I.	This paper	http://wrap.warwick.ac.uk/101835
*Salmonella* supertree 2 A species tree (ASTRID) based on 3,002 core gene trees from 2,964 representative genomes from *S. enterica* subsp. I.	This paper	http://wrap.warwick.ac.uk/101836
*Salmonella* supertree 3 A RAxML maximum likelihood phylogeny based on a 2.8 Mbp concatenate of 3,002 core genes from 2,964 representative genomes from *S. enterica* subsp. I	This paper	http://wrap.warwick.ac.uk/101837
SK152 reads: Non-human metagenomic data (SK152)	This paper	https://sid.erda.dk/wsgi-bin/ls.py?share_id=E56xgi8CEl
Workflow Workflow to reconstruct the Ragna genome	This paper	http://wrap.warwick.ac.uk/101838

**Software and Algorithms**		

TemporalFreq.py and data: A script for extracting rate of gene gain/loss and pseudogenes from the branches of an ML tree to generate data for Figure S3A,B	This paper	https://github.com/zheminzhou/TemporalCurve
PAIDB	[40]	http://www.paidb.re.kr/
ISfinder	[41]	https://www-is.biotoul.fr/
Concoct	[12]	https://github.com/BinPro/CONCOCT
Kraken	[42]	https://ccb.jhu.edu/software/kraken/
RAxML v8.2.4	[43]	https://sco.h-its.org/exelixis/software.html
Astrid	[44]	https://github.com/pranjalv123/ASTRID/
Beast 1.8.3	[45]	https://github.com/beast-dev/beast-mcmc/releases
LSD	[46]	http://www.atgc-montpellier.fr/LSD
BayesTraits	[47]	http://www.evolution.rdg.ac.uk/BayesTraits.html
OxCal 4.2	[48]	http://intcal.qub.ac.uk/intcal13/
BWA	[49]	http://bio-bwa.sourceforge.net/
Megahit	[50]	https://github.com/voutcn/megahit
Concorde	[51]	www.math.uwaterloo.ca/tsp/concorde.html
Prodigal	[52]	https://github.com/hyattpd/Prodigal
Mafft	[53]	https://mafft.cbrc.jp/alignment/software/
Trimal	[54]	http://trimal.cgenomics.org/
FastTree	[55]	www.microbesonline.org/fasttree/
MapDamage 2.0	[56]	https://ginolhac.github.io/mapDamage/
EnteroBase	[18]	https://enterobase.warwick.ac.uk/
Spades 3.5	[57]	http://spades.bioinf.spbau.ru/release3.5.0/
MGPlacer	[58]	https://sourceforge.net/projects/mgplacer/
BLAST	[59]	ftp://ftp.ncbi.nlm.nih.gov/blast/executables/blast+/
BBmerge & BBduk2 in BBmap	[60]	https://sourceforge.net/projects/bbmap/
Bowtie2	[61]	http://bowtie-bio.sourceforge.net/bowtie2/
SAMtools/BCFtools 1.2	[62]	http://www.htslib.org/
Last	[63]	http://last.cbrc.jp/
TRF	[64]	https://tandem.bu.edu/trf/trf.html
PILER-CR	[65]	https://www.drive5.com/pilercr/
RecHMM	[25]	https://github.com/zheminzhou/EToKi
Date randomization test	[38]	N/A
TempEst	[37]	http://tree.bio.ed.ac.uk/software/tempest/
ETE3 Python package	[66]	http://etetoolkit.org/
PROKKA	[67]	https://github.com/tseemann/prokka
UClust	[68]	https://www.drive5.com/usearch
APE package of R	[69]	http://ape-package.ird.fr/
PHASTER	[70]	http://phaster.ca/
PlasmidFinder	[71]	https://cge.cbs.dtu.dk/services/PlasmidFinder
CONJscan-T4SSscan	[72]	https://research.pasteur.fr/en/software/conjscan-t4ssscan/
Anvi’o	[73]	http://merenlab.org/software/anvio/
Sickle	[74]	https://github.com/najoshi/sickle
RASP	[75]	http://mnh.scu.edu.cn/soft/blog/RASP

**Other**		

Pan-Genome: Interactive Anvi’o Plot for the pan genome of the Para C lineage.	This paper	https://enterobase.warwick.ac.uk/anvio/public/zhemin/ParaC_pangenome
Para C lineage: Interactive 220 genomes in the Para C lineage plus 2 Birkenhead genomes	This paper	http://enterobase.warwick.ac.uk/species/senterica/search_strains?query=workspace:3246
Ragna: Ragna genome	This paper	http://enterobase.warwick.ac.uk/species/senterica/search_strains?query=workspace:3246
rST representatives: Interactive workspace for 2,964 genomes of one representative per ribosomal MLST ST in *Salmonella enterica* subsp. I	This paper	http://enterobase.warwick.ac.uk/species/senterica/search_strains?query=workspace:3247
SPI-6: Interactive Anvi’o Plot for the SPI-6 genomic island in the 221 genomes	This paper	https://enterobase.warwick.ac.uk/anvio/public/zhemin/ParaC_SPI6

### Contact for Reagent and Resource Sharing

Further information and requests for resources and reagents should be directed to and will be fulfilled by the Lead Contact, Mark Achtman (m.achtman@warwick.ac.uk).

### Experimental Model and Subject Details

Permission to undertake genetic analysis on SK152 was granted to MTP Gilbert by the Norwegian Research Ethics Committee (ref 2011/73).

#### SK152

SK152 was excavated in June, 1985, from grave 523E in a cemetery on the north side of a church in Trondheim, Norway (Figure 1A). The skeleton rested on a wooden plank (symbolic coffin) (Figure 1B). According to the daybook written in the field, the grave was filled with “grey, sandy loam, wood chips” which likely represents a mixture of minerogenic soil and waste from wood-building activities in the nearby, densely built-up area. The grave itself was covered with a wooden chip layer derived from later building activity in the area. The stratigraphical information indicates that the environment surrounding SK152 was anoxic, acidic, and waterlogged.

##### Preservation

The overall surface preservation of bone elements was very good, grade 1 according to the seven-category grading system defined by McKinley [76]. The skeleton was over 90% complete, with almost all bone elements present. Only minimal localized areas of bone damage due to post mortem disturbance were noted, and a small degree of fragmentation. Discoloration due to burial taphonomy was noted, but this was characteristic of the whole assemblage and did not impact on the visual inspection of bone surface.

##### Age and sex

Skeleton 152 was assigned to the young adult category (19-24 years) [77] on the basis of dental eruption and dental wear pattern, degeneration of the pelvis and fusion of epiphyses. Standard techniques for osteological sex determination [78] indicated that SK152 was female, and this conclusion was confirmed by the ratio of sequences aligning to the Y chromosome relative to the total number of sequences aligning to both sex chromosomes [79]. The mtDNA haplogroup was H4a.

##### Origins

The oxygen composition in enamel apatite carbonates from the first (M1) and third (M3) molars yielded M1 and M3 δ^18^O_Carbon_ values of 21.13‰ and 24.72‰ on the VSMOW scale, respectively. The δ^18^O_Carbon_ data was converted to δ^18^O_water_ (oxygen composition in water/precipitation) by using Equation 6 from citation [80] as modified by citation [81], to yield δ^18^O_water_ values of −15‰ to −16.5‰ for M1 and −9.3 ± 1‰ for M3 [14]. The values from the first molar suggest that SK152 was born inland in northern Scandinavia or in the north-western regions of Russia, whereas the values from the third molar indicate that she arrived in Trondheim in her childhood years [14].

### Method Details

#### Dating of SK152

The burial of SK152 was dated by two alternative methods (Figure S1). Grave 523E was part of sub-phase FN7/level II within an extension of the cemetery, corresponding to a building phase when the cemetery had recently been extended northward, and covered older/earlier buildings from sub-phase FN6. The construction of FN6 began ca. 1150 CE. Sub-phase FN7 was initially dated to “mid 1200s” with the aid of ceramics, coins, ^14^C-dating and archaeological lead artifacts (Reed in citation [82], p. 192), but this estimate was later revised to 1175-1225 CE on the basis of additional dendrographic dating (Figure 23 in citation [83]). Accelerator Mass Spectrometry (AMS) dating was performed at DirectAMS and Oxford Radiocarbon Accelerator Unit, yielding dates in radiocarbon years BP (Before 1950 CE) using the ^14^C half-life of 5,568 years. Isotopic fractionation was corrected using the δ^13^C values measured on the AMS. The quoted δ^13^C values were measured independently on a stable isotope mass spectrometer (to ± 0:3 per mil relative to VPDB). For details of the chemical pre-treatment, target preparation and AMS measurement see [84, 85]. Figure S1B shows calendar age ranges calculated by the OxCal computer program (v4.2) of C. Bronk Ramsey [48], using the ‘IntCal13’ dataset [86]. These calculations support dates of 994-1052 CE with 53% likelihood and 1081-1152 CE with 42% likelihood. We summarized these results as 1,073 ± 79 years, which is the mean ± range of the lowest and highest extremes.

#### Metagenomic sequencing of samples from SK152

All molecular work including pre-library amplification was conducted in dedicated aDNA clean laboratory facilities at the Centre for GeoGenetics, Natural History Museum, University of Copenhagen. All samples were collected and processed using strict aDNA guidelines. Nine sequencing libraries were prepared from the upper 3^rd^ left molar root including dental pulp, the upper 2^nd^ right molar dentine/cementum (200 mg) and pulp (interior root canal, 200 mg), femoral long bone (300 mg), and a mixture of mineralized dental plaque (calculus, 30 mg) taken from multiple teeth. These samples were processed in a specialized drill room within the dedicated aDNA facilities.

In the initial investigation, the entire root of the upper 3^rd^ left molar was crushed, and DNA extracted according to Rohland and Hofreiter [87], with a pre-digestion step as described by Allentoft et al. [88]. The same protocol was used for the interior dental pulp. For the upper 2nd right molar, the entire tooth was removed from the mandible, and the tooth crown separated horizontally from the tooth root with a diamond-dust-coated cutting disk in a mechanical drill. The tooth root surface was then cleaned with a new cutting disk before using a small pointed drill bit to remove the interior dental pulp. DNA was extracted from this pulp as above. The remaining dentine and cementum fractions were crushed with a hammer, and also extracted. (We extracted dentine as well as cementum because this has previously maximized the yield of endogenous DNA.) The extraction protocol for this material and for dental calculus fully followed the protocols of Allentoft et al. [88] which are based on silica powder-based extraction, except that silica powder was only incubated for 1 h in the supernatant rather than the full 3 h.

DNA libraries for sequencing were prepared through blunt end ligation using NEBNext DNA sample preparation reagents (E6070) and Illumina specific adapters following established protocols. The libraries were shotgun sequenced (Table 1) in pools across 15 different lanes using Illumina HiSeq 2500 and 4000 platforms, and with a mix of 80 and 100-bp single read and 150-bp paired end chemistry. All pools submitted for sequencing contained between 5-15nM of DNA.

After initial screening, the highest proportion of *Salmonella*-specific reads were found in the upper 2nd right molar dentine/cementum (Table 1). Additional libraries were constructed from the same extracts with the same protocol, and also shotgun sequenced to increase the depth of coverage.

#### Genomic assemblies from metagenomic sequences of samples from SK152

##### Genomic assemblies from metagenomic reads

Taxonomic profiling of metagenomic reads with currently available traditional methods can yield false indications of the presence of bacterial pathogens because pathogens are over-represented in public databases relative to environmental organisms, and completed genomes of many environmental bacteria are not yet available. As a result, ecological metagenomic analyses have used *de novo* assemblies of raw reads [89] but this has not yet been applied in aDNA studies. We therefore investigated whether *de novo* assemblies could reveal the presence of bacterial pathogens within the nine metagenomic libraries from SK152, and used subsequent analysis of deamination rates to determine whether those assemblies were ancient or modern.

##### Assembly and binning

We excluded reads within the metagenomic libraries that mapped to the human genome according to BWA [49]. The non-human reads were co-assembled into contiguous sequences (contigs) with MEGAHIT [50], using default parameters. Contigs which exceeded 20 kb were split into 10 kb fragments. All 39,016 contigs greater than 1 kb in length were clustered by Concoct [12] into 76 bins using both sequence composition and coverage across all samples. All of the contigs in each bin were potentially derived from a single bin-specific genomic assembly. Protein encoding sequences on these contigs were called using Prodigal [52], and then categorised in terms of function according to Clusters of Orthologous Groups of proteins (COGs) [90]. We previously identified 36 single copy core COGs (SCGs) that are found in a single copy in all bacterial genomes [12]. We therefore tested each of the bins for the number of copies of these 36 SCGs, and found that 11 bins largely represented MAGs (unique metagenome assembled genomes) because they contained at least 27 SCGs (75% of 36) in a single copy.

##### Taxonomic assignments

We constructed a phylogenetic tree of the 36 SCGs from 1,755 reference genomes plus the 11 MAGs. The reference genomes consisted of one representative from each bacterial genus and each archaeal species with complete genome sequences in NCBI. Each SCG was aligned separately using MAFFT [53], and overhangs were trimmed with TRIMAL [54]. Where exceptional MAGs contained multiple SCGs, we chose one of the sequence variants at random. The concatenated SCG alignments were used to construct a single tree with FastTree [55]. For taxonomic assignments, we identified the ancestral node containing each MAG plus one or more reference genomes, and the most frequent taxonomic designation among the neighboring reference genomes was assigned to that MAG (Concoct 3, see Key Resources Table). C72 was initially assigned to *Mogibacterium timidum*, but only few related reference genomes existed in NCBI. We therefore compared C72 against the rMLST database [91] which contains > 200K genomes representing over 6,000 bacterial species. To this end, the translated amino acids of representative alleles in the rMLST database were mapped onto C72 using tBLASTn. This identified the positions of 46 of the 53 ribosomal genes. Those 46 ribosomal genes were then compared against all genomes in the rMLST database using tBLASTn. Analyses of both concatenated sequences and gene-by-gene analyses showed that C72 was more similar to *Eubacterium sulci* (74% identity) than *Mogibacterium timidum* (70%). We therefore re-assigned C72 to *Eubacterium.*

##### Calculation of deamination rates

BWA was used to map the metagenomic reads onto all contigs within each MAG, as well as to the human genome hg38 and Paratyphi C RKS4594. We then used mapDamage 2.0 [56] to separately characterize the DNA damage associated with each of the 13 organisms. The posterior mean estimates and standard deviations for δS, the single-stranded deamination rate for all genomes and MAGs (Concoct 3, see Key Resources Table) are depicted graphically in Figure 1D.

##### Interpretation of sources of MAGs

According to Kistler *et* al. [15], nine of the MAGs likely reflect recent soil contamination because their 5′-single-stranded deamination rates were low (DNA damage < 0.22) versus values of 0.47 for the human DNA and 0.9 for Ragna (Concoct 4, see Key Resources Table). C69 was unambiguously classified as *Methanosphaerula palustris*, a archaeal methanogen which is associated with acidic peat bogs [92], and belongs to a group of hydrogenotrophic methanogens with no human-associated relatives. Similarly, C40 and C66 were assigned to the environmental sulfate-reducing species *Desulfatiglans anilini* and *Desulfomonile tiedjei,* respectively, but not to human-associated sulfate reducers, such as *Desulfovibrio* species [93].

Two other assembled genomes exhibited high levels of DNA damage. C72, a novel species of *Eubacterium*, may have been a major component of a biofilm associated with periodontal disease [16]. In support of this role, C72 was recovered almost exclusively from dental calculus whereas reads from the putative environmental taxa were present in multiple sources (Concoct 5, see Key Resources Table). C18 belongs to *Acidovorax*, which is associated with plant pathogens [17], and may have been introduced with the wood chips that covered the skeleton.

#### Genotyping by EnteroBase

For selected genera, EnteroBase (see Key Resources Table) automatically assembles genomes from all publicly available Illumina short reads as well as from short reads that are uploaded by users [18]. *De novo* assemblies of Illumina reads are superior to reference based mapping because they recover accessory genomic regions which are not necessarily present in the reference genome. However, all current assemblers yield a certain proportion of false base calls which do not necessarily represent the consensus of all reads. EnteroBase therefore implements a post-assembly pipeline to call SNPs based on the consensus. In brief, the ends of sequenced reads with base quality less than 5 are removed (trimmed) using Sickle [74, 94] and assembled into contigs using SPAdes 3.5 [57] with the parameter ‘–only-assembler’ and k-mers equal to 0.3, 0.5, 0.7 and 0.9 of the average read lengths. To validate the consensus call for each base in the assemblies, the original trimmed, sequenced reads are mapped back to the corresponding assembled contigs using BWA [49], and analyzed with SAMtools/BCFtools 1.2 0.7.12-r1039 [95]. The quality scores for consensus calling for each base are stored together with the assemblies in standard FASTQ format. Finally, assembled contigs are assigned taxonomic labels using Kraken [42] in order to exclude potential contamination from other genera.

Assemblies that pass internal quality criteria, including a mean coverage of ≥ 20-fold are automatically genotyped by multiple multi-locus sequence typing (MLST) schemes into Sequence Types (STs) consisting of unique allele numbers for each genetic locus. Details of these schemes are available on the Help pages at EnteroBase. For *Salmonella*, these MLST schemes currently include a 7 housekeeping gene legacy scheme [3] and rMLST based on 51 ribosomal proteins as defined by Jolley et al. [91], and whose reference allelic sequences are maintained at the primary rMLST database at Oxford University [91]. EnteroBase also calculates alleles for wgMLST based on a pan-genome of 21,065 genes. In brief, the wgMLST scheme encompasses unique sets of homologs with ≥ 70% pairwise amino acid similarity over 50% of their length, and which were defined on the basis of 537 diverse, high quality *Salmonella* genomes. These homolog sets are usually either absent from individual *Salmonella* genomes, or are present only in a single copy. cgMLST (core genome MLST) V2 consists of that subset of 3,002 loci from the wgMLST scheme which met the following conditions for the 3,144 rMLST STs in *S. enterica* that had been defined by May, 2016: presence in ≥ 98% of genomes; an intact reading frame in ≥ 94%; and of unexceptional genetic diversity. For several homolog sets, more than one copy is occasionally present per genome. When rare genomes contain two or more copies of any wgMLST or cgMLST locus, that locus is scored internally as duplicated for that genome, and is hidden to public access for that genome.

##### Species trees of *S. enterica* subspecies I

Most aDNA analyses have been performed with genetically monomorphic pathogens with only limited genetic diversity, and where recombination is rare. However, typical species containing bacterial pathogens, such as *S. enterica,* are usually much more genetically diverse, and commonly also undergo recombination. Until now a reliable and publicly available phylogenetic topology of *S. enterica* genomes did not exist, and it was not readily possible to accurately assign individual metagenomic reads to individual lineages [96]. We therefore assembled a collection of genomes that represent the entire genetic diversity of 50,000 genomes of *S. enterica* subsp. I by choosing one random representative of each of the 2,964 rMLST STs from that subspecies that were present in EnteroBase (May, 2016) (*Salmonella* supertree 1, see Key Resources Table). These genomes can be accessed at the Enterobase Workspace rST Representatives (see Key Resources Table). We performed super-tree analyses on the sequences of the 3,002 core genes in cgMLST v2 from these 2,964 representatives. The sequences of each core gene were aligned using Mafft [53]. These alignments were used to calculate species trees by two different algorithms. i) A Maximum Likelihood tree of the concatenated, aligned sequences of all core gene sequences (2.8 Mb) (*Salmonella* supertree 3, see Key Resources Table) was generated using a GTRCAT model in RAxML v8.2.4 [43] (Figure 2A). ii) Maximum Likelihood trees were also generated by RAxML for each of the 3,002 core gene alignments, and these 3,002 gene trees were processed by Astrid [44]. Astrid is a recently developed coalescent-based method for species tree estimation that is statistically consistent under the multi-species coalescent, can account for incomplete lineage sorting, is one of the most accurate currently available methods, and can handle large amounts of data. Because Astrid only infers topology, we derived the branch lengths from sequence distances within the concatenated alignment using RAxML with a GTRGAMMA model (*Salmonella* supertree 2, see Key Resources Table). The topologies of the two trees were identical near the root and toward the tips, and at 75% of the intermediate branches. These trees were used for phylogenetic placement (MGPlacer) [58] of the metagenomic reads from SK152. We also compared the results from Astrid with those from a separate species tree (data not shown) calculated with the help of a GPU compilation of Astral-II [97] by S. Mirarab and T. Warnow. The species trees generated by Astral and Astrid were almost indistinguishable (data not shown). In all three species trees, two genomes from the rare serovar Birkenhead clustered unambiguously with the Para C lineage, and were used as an outgroup for rooting trees in further analyses (Figure 2).

#### Sources of genomes from the Para C Lineage

The species trees identified 100 genomes in Enterobase that were in the Para C lineage, or closely related to it. Most of these genomes were very recent, epidemiological isolates from the UK (PHE) and USA (FDA), but a number of them were from strains sequenced by the Wellcome Trust Sanger Centre that had been tested by legacy MLST [3] or were from the historical Murray collection [98]. In order to ensure that we spanned diverse sources and dates, we cultivated 119 additional old isolates from the historical, global collection at the Institut Pasteur, Paris, and also sequenced their genomes.

Total DNA was extracted with a Maxwell 16 cell DNA purification kit (Promega, Madison WI), in accordance with the manufacturer’s recommendations. Libraries were constructed using the Nextera XT kit (Illumina) with the following modifications. The initial tagmentation reaction was performed using 2 μl of 0.7 ng/μl of template DNA and 2/5 of the specified volume for other reagents, resulting in a volume of 10 μl after neutralization. For the PCR step, 25 μl of 2x Extensor Hi-Fidelity PCR master mix (Thermo Scientific), 5 μl of each index primer (4 μM) and 5 μl of sterile distilled water was added to the tagmentation reaction. The standard PCR reaction was extended by an extra 3 cycles, and the extension step was lowered from 72°C to 68°C. The libraries were purified using 25 μl of Ampure XP beads (Beckman Coulter) with two 200 μl washes with 80% ethanol before elution in 30 μl of RSB from the Nextera XT kit. Libraries were quantified using the Qubit dsDNA HS Assay Kit (Thermo Scientific), and diluted to 3.2 ng/μl (approx. 8 nM). Pooled libraries (40 samples per run) were denatured following the Illumina protocol, and 600 μl (approx. 20 pM) was loaded onto a MiSeq V2 −500 cycle cartridge (Illumina), and sequenced on a MiSeq to produce FASTQ files of short reads.

The short reads were uploaded to EnteroBase and to the Short Read Archives. Their metadata and genome properties are summarized together with other genomes of the Para C lineage in Table S5 and can be examined interactively in the EnteroBase public workspace Para C lineage (see Key Resources Table).

#### *Salmonella* short reads from metagenomic aDNA

##### Initial analyses

Metagenomic reads from 33 human skeletons from Trondheim, Norway were screened using Kraken [42] with its default genomic database (Workflow, see Key Resources Table). Kraken scored 304 metagenomic reads from skeleton SK152 as being *Salmonella,* and did not score any reads from the 32 other skeletons as *Salmonella* or other potentially invasive bacterial pathogens. However, the taxon assignments provided by Kraken can be problematical when reads are present at low concentrations [99]. In our hands, the Kraken analyses of SK152 also failed to identify seven of the eleven taxa that yielded assembled MAGs with Concoct (Concoct 6; Concoct 7, see Key Resources Table). We therefore tested the reads from SK152 that had been identified as *Salmonella* by *ad hoc* BLASTn alignments to a custom database based on 91,000 bacterial, archaeal, and viral genomes from the non-redundant nucleotide database in GenBank, and confirmed that 266 (87%) scored as *S. enterica*. MGPlacer [58] was then used to iteratively map those *Salmonella*-specific metagenomic reads onto a core SNP phylogeny based on one genome from each of the 20 *Salmonella* serovars represented in RefSeq (May, 2016). All *Salmonella*-specific reads mapped on the branch leading to serovar Paratyphi C strain RKS4594.

##### Ragna-specific reads in metagenomic libraries

Reads were pre-processed using BBmerge and BBduk2 from the BBmap package [60]. Sequences specific to the Ragna genome were then identified by alignment against two sets of reference genomes using Bowtie2 [61]: i) The “ParaC” set, consisting of all 219 modern genomes in the Para C lineage and ii) the “outgroup” set, consisting of representatives of all (4,441) non-*Salmonella* bacterial genomes in RefSeq, plus the human genome hg38. We assigned sequencing reads to Ragna if they yielded equal or better alignment scores to the ParaC set than to the outgroup, and differed in sequence from the most similar genome in the ParaC lineage by no more than 4%. Potentially duplicated reads were removed by retaining only one Ragna-specific read sequence when multiple identical reads were identified. The quality of the Ragna-specific reads are summarized in Figure S2.

##### SNPs in the Ragna genome

The Ragna-specific, unique reads were aligned against Paratyphi C reference genome RKS4594 using Bowtie2 [61] with the end-to-end option and analyzed using SAMtools/BCFtools 1.2 [62]. In order to exclude potential spurious SNPs due to deamination (Figure S2C), the consensus base was only called on sites that were covered by at least two reads with a consensus base quality ≥ 10 and which were located at least 5 bases from either read end (Figure S2B). Exceptionally, SNP calls with single coverage matching these criteria were included if the same call was provided by previously excluded 5′ and 3′ ends of reads.

##### Reference based SNP calling in modern genomes

Assemblies within the Para C lineage plus Birkenhead (Figure S4) were aligned against RKS4594 using Last [63], and SNPs from these alignments were filtered to remove regions with low base qualities (Q < 10) or ambiguous alignment (ambiguity ≥ 0.1). Sites were also removed if they aligned with ≥ 95% identity to disperse repetitive regions that were longer than 100 bp (BLASTn), overlapped with tandem repeats (TRF) [64], or overlapped with CRISPR regions (PILER-CR) [65]. Of the remaining SNPs, 61,451 were called in ≥ 95% of the genomes from the ParaC Lineage plus Birkenhead, and were retained as core genomic SNPs (ParaC genome SNPs 2, see Key Resources Table).

##### Reconstruction of the mutational phylogeny of the Para C lineage

An initial phylogeny (ParaC genome SNPs 3, see Key Resources Table) was calculated on all 61,451 core SNPs from the Para C lineage plus Birkenhead using RAxML v8.2.4 [43] under a GTRCAT model with Stamatakis ascertainment correction for invariant sites [100]. SNPs were assigned using RecHMM [25] onto branches in that phylogeny using a maximum likelihood method with a symmetric transition model [101]. The results indicated that 21,071 genomic sites had suffered substitution events along the branches leading to serovars Paratyphi C, Typhisuis and Choleraesuis, of which 406 sites (1.9%) were mutated on multiple independent occasions (homoplasies) resulting in a total of 21,849 substitution events (ParaC genome SNPs 1; ParaC genome SNPs 2, see Key Resources Table). RecHMM also identified 1,602 SNPs that were clustered, which are the hallmarks of homologous recombination. After excluding the recombinational SNPs, the remaining 20,247 core mutational SNPs were used for further phylogenetic analyses. The RAxML phylogeny based on these mutational SNPs (Figure 2B) differed only in branch lengths but not in topology from the initial RAxML phylogeny based on all core SNPs. The mutational phylogeny was also used for additional analyses of gene gain and loss and numbers of pseudogenes (Figure 3; Para C Pan-genome 1, see Key Resources Table) and ancestral reconstruction (Ancestral reconstruction 1, see Key Resources Table).

In separate experiments, recombinant SNPs on the branches to serovars Paratyphi C, Typhisuis and Choleraesuis were called by the alternative programs ClonalFrameML [102] and Gubbins [103] and compared with the calls by RecHMM. 20,023 SNPs were scored as mutational and 1,407 SNPs as recombinational by all three programs, but the programs differed in their assignments for 398 SNPs (1.8%) (ParaC genome SNPs 4, see Key Resources Table). In contrast, the three programs differed dramatically in their assignments of SNPs on the branches to Lomita and Birkenhead, and we did not attempt to subdivide those SNPs into recombinational versus mutational.

#### Date estimates for Paratyphi C and related serovars

Date estimates based on Bayesian phylogenetic analysis are summarized in Table S4.

##### Bayesian phylogenetic approach

Core mutational SNPs were analyzed using BEAST v1.8.3 [45] with the GTR+G model of nucleotide substitution, with a discrete gamma distribution and six rate categories to account for rate heterogeneity across sites. To account for SNP ascertainment bias, we applied a correction that incorporated the nucleotide frequencies across all of the constant sites. For computational tractability, we analyzed two subsamples each from the Para C lineage without Lomita; serovar Paratyphi C serovar (including Ragna); and modern crown serovar Paratyphi C strains (excluding Ragna). Each subsample comprised 50 random genomes, except that the Ragna sequence and the sole genome in the CS-3 cluster were deliberately included when present in the test set. Sub-samples including the Ragna genomes were run with either of two dates for Ragna (1200 CE from archaeological dates and 1073 CE from AMS calibrated ages). Thus, our Bayesian phylogenetic analyses involved 12 datasets (Date estimation 3, see Key Resources Table). The raw trees are presented in Date estimation 4 (see Key Resources Table).

Posterior distributions of parameters, including the MCC tree, were estimated using Markov chain Monte Carlo sampling. Samples were drawn every 5,000 steps over a total of 5 × 10^7^ steps, with the first 10% of samples discarded as burn-in. Marginal likelihoods were used to compare two clock models (strict clock and uncorrelated lognormal relaxed clock) and three demographic models for the tree prior (constant population size, Bayesian Skyride coalescent [104], and birth-death process with serial sampling [105]). Marginal likelihoods were estimated using stepping-stone sampling [106], with 20 path steps and a chain length of 10^6^ per path step, and the most likely model combinations are indicated in bold print in Date estimation 3 (see Key Resources Table). This table also presents the median MRCA estimates plus 95% credible intervals based on all six model combinations for the Para C lineage, Paratyphi C including Ragna and the Paratyphi C modern crown lineage (without Ragna). The optimal model for Paratyphi C including Ragna was the strict clock model with constant population size. The optimal model for the ParaC Lineage was UCLD (uncorrelated relaxed clock) model with constant population size. To ensure that these results did not represent outliers due to limited numbers of runs, we also tested eight additional subsamples using these optimal models and assuming a date for Ragna of 1200 CE. The median values for their estimates of tMRCAs and mutational clock rates and the corresponding 95% confidence intervals are summarized in Table S4.

##### Temporal structure

It is crucial to verify the existence of temporal structure in datasets used for date estimation. We therefore tested the reliability of the Beast analyses by date randomization tests [38] which analyze multiple, date-randomized replicate datasets after randomly reassigning the ages of the sequences. Datasets are considered to have strong temporal structure when the 95% credible interval of the rate estimate from the original data does not overlap with those of the rate estimates from the date-randomized replicates [107]. We used 10 date randomizations for each of the initial two sub-samples, with and without Ragna, and found strong temporal signals within all sub-samples of modern genomes analyzed with Beast (Date estimation 1, see Key Resources Table).

##### Root-to-tip regression

We also used an alternative method to estimate temporal signal for dating on the non-recombinational SNPs in 116 Paratyphi C genomes and the 205 Para C lineage genomes with known collection dates, including Ragna but excluding Lomita. This consisted of a regression of root-to-tip distances with TempEst [37] (previously designated Path-O-Gen) in which the degree of within-lineage rate heterogeneity is evaluated by calculating the correlation coefficient. A regression based on one aDNA genome (Ragna) plus multiple modern genomes is basically a regression line based on two points. We therefore also included two additional aDNA genomes, Tepos-14 and Tepos-35 from 1,545 CE in Mexico [19]. The results (Data estimation 2, see Key Resources Table) showed a clear temporal signal in both datasets, with an extrapolation to the root which was similar to the tMRCAs found with Beast. The Paratyphi C dataset yielded R^2^ of 0.38, which confirms the existence of temporal signal. The Para C lineage only had an R^2^ of 0,04 indicating that TempEst is not suited for the analyses of these data.

#### Inferring ancestral geographic sources

A subtree of genomes with geographic source information was extracted from the Para C lineage phylogeny plus the outgroup Birkenhead with the ETE3 Python package [66]. (Lomita is lacking from this subtree because its geographic source is uncertain). The ancestral geographic states of internal nodes in the subtree were inferred by three independent algorithms: i) the Maximum Likelihood comparison [108] in BayesTraits [47]; ii) the Markov chain Monte Carlo (MCMC) approach [109] in BayesTraits; and iii) Bayesian Binary MCMC in RASP [75]. All three algorithms indicated a European origin for individual nodes within the Para C lineage (Ancestral reconstruction 1; Ancestral reconstruction 2, see Key Resources Table), and this conclusion was not affected by the inclusion or absence of Ragna. A European origin of the entire Para C lineage was also strongly supported by BayesTraits MCMC and RASP, and moderately supported by BayesTraits ML (Ancestral reconstruction 2, see Key Resources Table).

#### Reconstruction of the pan genome of the Para C lineage

A wide collection of annotated reference genes was collected from three sources: 1) all 21,065 unique sets of homologs in the wgMLST scheme in Enterobase; 2) published annotations for the complete genomes of Choleraesuis strains C500 [110], SC-B67 [111] and Paratyphi C strain RKS4594 [112]; 3) Prokka [67] annotations of all draft genomes in EnteroBase. All these reference genes were grouped into 29,436 gene clusters using UClust [68]. In order to obtain sets of homologous regions that cover ≥ 50% of the length of the centroid sequences with ≥ 70% nucleotide identity, the centroid sequence from each cluster was aligned with all the modern genomes in the Para C lineage using BLASTN. Overlapping paralogs between the homolog sets were identified through the same iterative methodology used to construct the entire *Salmonella* wgMLST scheme. After removal of paralogs, the remaining 6,665 homolog sets were treated as the pan genes of the Para C lineage (ParaC Pan-genome 4; ParaC Pan-genome 5, see Key Resources Table).

##### Reconstruction of pan genome synteny

Synteny was reconstructed as described in Supplemental Materials of Zhou et al. [26], namely through constructing and traversing a graph of assembled sequences from genes in the Para C lineage pan-genome. First, the graph was seeded with one node for each unique, single copy gene. The connections between nodes were weighted using the following criteria: 1) Edges connecting pairs of genes that were co-located on a single contig received maximal weighting. 2) Edges received intermediate weighting that connected two genes at the ends of distinct contigs that were however linked by read-pairs that straddled both contigs. This intermediate weighting was 2^∗^(number of read-pairs joining the two contigs)/(total number of unpaired reads at the ends of contigs). 3) Pairs of genes which did not co-locate according to either of these two criteria were not assigned to a common edge.

Concorde [51] was used to find the shortest possible path that visited all the nodes in the graph, which equates to the most likely gene order within the Para C lineage. Ambiguous paths were inspected manually to identify duplicated genes and collapsed repeats, because these are usually associated with prophages or plasmids. Such connections were then broken manually and re-joined as appropriate. Finally, in order to reconstruct the synteny of the pan genome of the Para C lineage, all repetitive genes were inserted into the gene order according to their location within the assemblies (Figure 2B and ParaC Pan-genome 4, see Key Resources Table). A total of 227 genomic islands (ParaC Pan-genome 1; ParaC Pan-genome 7, see Key Resources Table) were identified as continuous blocks of gene gain/loss in the pan genome of the Para C lineage.

The conservation of genes within the pan genome across the Para C lineage was illustrated using Anvi’o [73] (Figure 2B), and can be examined in detail in a publicly accessible, interactive EnteroBase version of Anvi’o (Pan-genome, see Key Resources Table). The synteny of pan genes was enforced within the Anvi’o rendering by help of an artificial guide tree based on the gene order in ParaC Pan-genome 4 (see Key Resources Table), where each sequential gene bifurcates from the previous gene at a constant distance. (That artificial tree was deleted from the printed version in Figure 2B). The figures are dependent on manually generated input files based on the annotations and sub-divisions that are indicated in ParaC Pan-genome 4 (see Key Resources Table). The input files also include the frequencies of pan genes in all sub-lineages, the locations of major genomic islands, as well as additional metadata. These input files can also be downloaded from the interactive EnteroBase version.

##### Gain and loss of pseudogenes and intact genes

The gene alignments used to reconstruct the pan genome of the Para C lineage were screened for potential gene disruptions. Genes with stop codons or frameshifts anywhere in the coding regions were scored as pseudogenes. Pseudogenes and genes that had been gained or lost were assigned to ancestral states in the core SNP phylogeny (ParaC Pan-genome 1, see Key Resources Table) by the Maximum Likelihood algorithm [101] as implemented in the R function ACE (Ancestral Character Estimation) within the APE (Analyses of Phylogenetics and Evolution) package (ParaC Pan-genome 6; ParaC Pan-genome 7, see Key Resources Table) [69]. These ancestral states were used to infer the phylogenetic branches on the SNP tree of the Para C lineage on which 3,125 genes or parts of genes and 1,251 pseudogenes were independently gained or lost (Figure S3; ParaC Pan-genome 1; ParaC Pan-genome 3, see Key Resources Table). The relative frequencies of gain/loss of intact genes and pseudogenes versus time were estimated as described [25]: time (years) was calculated from the core SNP phylogeny by dividing branch lengths by the median SNP substitution rate estimated by BEAST (7.9 × 10^−8^ substitutions per site per year; Table S4). The 95% confidence intervals for these frequencies of pseudogenes and gene gain/loss were inferred from 1,000 separate bootstrap re-samplings of pseudogenes or genomic islands. The data and script used for these purposes can be found in TemporalFreq.py and Data (see Key Resources Table).

##### Identification of bacteriophages

Prophages in modern genomes of the Para C lineage were identified using the online API in PHASTER [70]. The identified prophages were taxonomically labeled according to the similarities of their major capsid protein (MCP) to the clusters of bacteriophages described by Casjens et al. [113]. Further manual taxonomic refinements were based on comparisons of all genes in each prophage against those in the published phage clusters. Prophage names are the same as the previously described phages which they most closely resembled, except for GI28, which has no known close relatives, and SPC-P1, which had been previously designated under that name in the annotation of Paratyphi C RKS4594 [28].

##### Annotation of genomic islands

BLASTN was used to identify known genomic islands within the pan genome as tight clusters of genes in which ≥ 60% of the sequences aligned with a previously described SPI/SGI sequence with ≥ 80% nucleotide identity. The genomic sequences used as references for SPI-1 through SPI-12 were downloaded from PAIDB [40], as were SGI-1 and SGI-2. SPI-13 through SPI-21 were obtained from citations [114, 115, 116, 117, 118].

Genes in the pan genome were also screened for an association with mobile elements that are listed in ISfinder (IS elements) [41], PlasmidFinder 1.3 (incompatibility groups of plasmids) [71] and CONJscan-T4SSscan (relaxases and key components of type IV secretion systems) [72]. Strong matches were annotated according to those resources (Figure 2B; ParaC Pan-genome 4; ParaC Pan-genome 7, see Key Resources Table). These analyses identified 227 genetic islands belonging to 127 distinct categories in which one or more genes were gained or lost (ParaC Pan-genome 3, see Key Resources Table).

### Quantification and Statistical Analysis

No statistical methods were used to predetermine sample size. The experiments were not randomized except for Bayesian analyses and the investigators were not blinded to allocation during experiments and outcome assessment.

### Data and Software Availability

Genomic reads for 119 strains from the Institut Pasteur collection have been deposited in the NCBI short read archive under project accession GenBank: PRJEB19916. All genomes referred to here are available from the publicly available workspaces entitled “rST representatives” (2,964 genomes) and “Para C lineage” in the *Salmonella* database of EnteroBase (http://EnteroBase.warwick.ac.uk). Interactive versions of Figure 2 and Figure S4 can be found at https://enterobase.warwick.ac.uk/anvio/public/zhemin/ParaC_pangenome and https://enterobase.warwick.ac.uk/anvio/public/zhemin/ParaC_SPI6. Additional data, figures, and tables are permanently stored at the University of Warwick and non-human metagenomic data at https://sid.erda.dk/wsgi-bin/ls.py?share_id=E56xgi8CEl and can also be accessed using the links provided in the Key Resources Table.

## Supplemental Information

Document S1. Figures S1–S4 and Tables S1–S4

Table S5. Detailed Metadata and Genome Statistics for 220 Bacterial Strains in the Para C Lineage plus 2 Birkenhead Strains, Related to Figure 2

Document S2. Article plus Supplemental Information
