# Supplementary material for: Pan-genome Analysis of Ancient and Modern Salmonella enterica Demonstrates Genomic Stability of the Invasive Para C Lineage for Millennia
Source: Curr Biol. 2018 Aug 6;28(15):2420–2428.e10. doi: 10.1016/j.cub.2018.05.058 (PMC6089836; doi:10.1016/j.cub.2018.05.058)
Supplement: Document S1. Figures S1–S4 and Tables S1–S4 [file mmc1.pdf]

Current Biology, Volume *28*

Supplemental Information

Pan-genome Analysis of Ancient and Modern *Salmonella enterica* Demonstrates Genomic Stability of the Invasive Para C Lineage for Millennia

Zhemin Zhou, Inge Lundstrøm, Alicia Tran-Dien, Sebastián Duchêne, Nabil-Fareed Alikhan, Martin J. Sergeant, Gemma Langridge, Anna K. Fotakis, Satheesh Nair, Hans K. Stenøien, Stian S. Hamre, Sherwood Casjens, Axel Christophersen, Christopher Quince, Nicholas R. Thomson, François-Xavier Weill, Simon Y.W. Ho, M. Thomas P. Gilbert, and Mark Achtman

A

Skeleton number	SK152
Sex	Female
Age (y)	19-24
Height (cm)	154 ± 3
Date (Archeology)	1200 ± 25 CE
AMS result (Oxford)	1073 ± 79 CE
AMS result (Direct AMS)	911 ± 30 CE
possible birth place ($\delta^{18}O_{Carbon}$ from first molar)	northernmost inland areas of Scandinavia/ Northwest Russia
travel history ($\delta^{18}O_{Carbon}$ from third molar)	travelled south at age of 3-14 and arrived in Trondheim in early teens

B

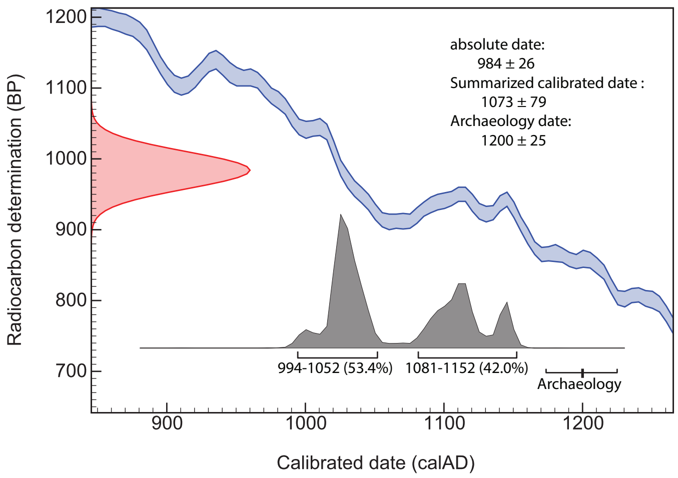

Figure S1. Archaeological findings for SK152. Related to Table 1. A) Summary of detailed information for SK152. B) Graphic illustration of the conversion between radiocarbon (^{14}C) dating (Oxford) and calibrated dates plus archaeological dating. The red curve on the Y axis shows the uncalibrated ^{14}C estimate of years before present. The blue line is the calibration curve, and the grey curves on the X-axis are the calibrated dates according to that calibration curve. The summarized calibrated date is the mean value of the extremes of the calibrated ^{14}C dates with a confidence interval of 79 years representing those extremes. Archaeology date: age of SK152 inferred from archaeological interpretations of the building phase of the cemetery.

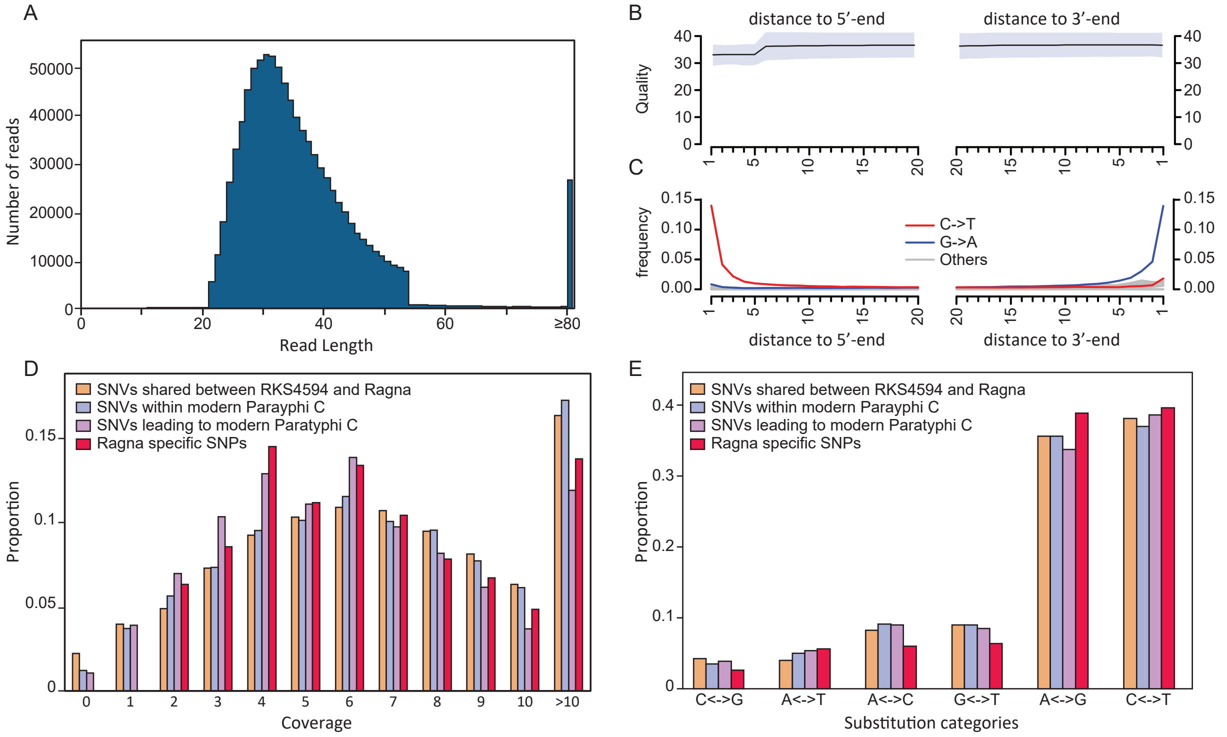

Figure S2. Properties of aDNA reads from SK152 that mapped to the genome of Paratyphi C (RKS4594). Related to Figure 1. (A-C) and properties of SNPs called in Ragna (D, E). A) Histogram of the read length distribution. The spike at 81 bps resulted because the reads were from single-end sequencing libraries. B) Base qualities of the reads in the terminal 20 bp at both ends (black lines: average quality; gray shadow: standard deviation). C) The deamination rate according to Mapdamage at both ends of the mapped reads. D) Histogram of read coverage by category of Single Nucleotide Variants. E) Histogram of nucleotide substitutions by category of Single Nucleotide Variants.

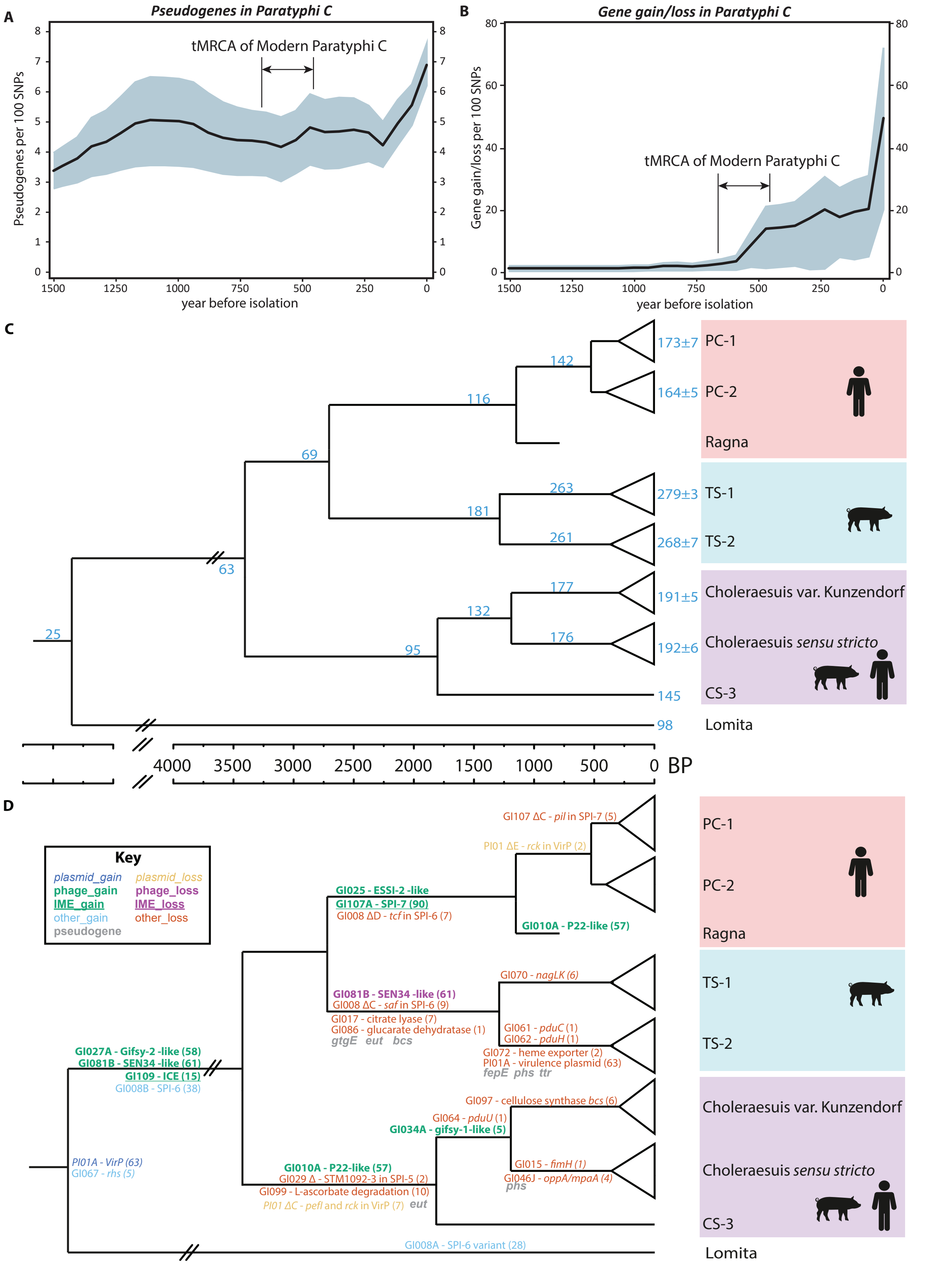

Figure S3. Accumulation of pseudogenes and gene gain/loss in Paratyphi C since its tMRCA. Related to Figure 3. (A) Accumulation of pseudogenes *versus* time. (B) Gene gain/loss versus time. A-B) The time range (years before isolation) was calculated for 50 year windows by multiplying branch lengths from a tip-based maximum-likelihood phylogeny by 7.9 x 10^{-8} substitutions per site per year. This was the mean substitution rate estimated by BEAST (Table S4). The data were generated using the script TemporalFreq.py. The black lines represent the mean frequencies of genetic events per 100 SNPs after random resampling (1,000 bootstraps) and shaded areas show CI95% estimates of the mean. (C) Cartoon core SNP tree of the sub-lineages of the Para C Lineage labelled on each branch with the numbers of accumulated pseudogenes. The mean numbers ±standard deviation within the modern genomes of each sub-lineage are indicated at the right of the collapsed triangles These data were extracted from ParaC Pan-genome 6. (D) Cartoon tree of the gain or loss of mobile elements that might potentially affect the phenotypic properties of the sub-lineages within the Para C Lineage extracted from ParaC Pan-genome 7. Numbers in parentheses: numbers of genes affected by each change. Partial deletions in PI01 (virulence plasmid VirP) and GI008 (SPI-6) are indicated by "Δ" followed by letters for distinct deletions. Key, Color code for types of GIs and gain/loss. C, D) Shaded areas at right: Sub-lineages and host specificity.

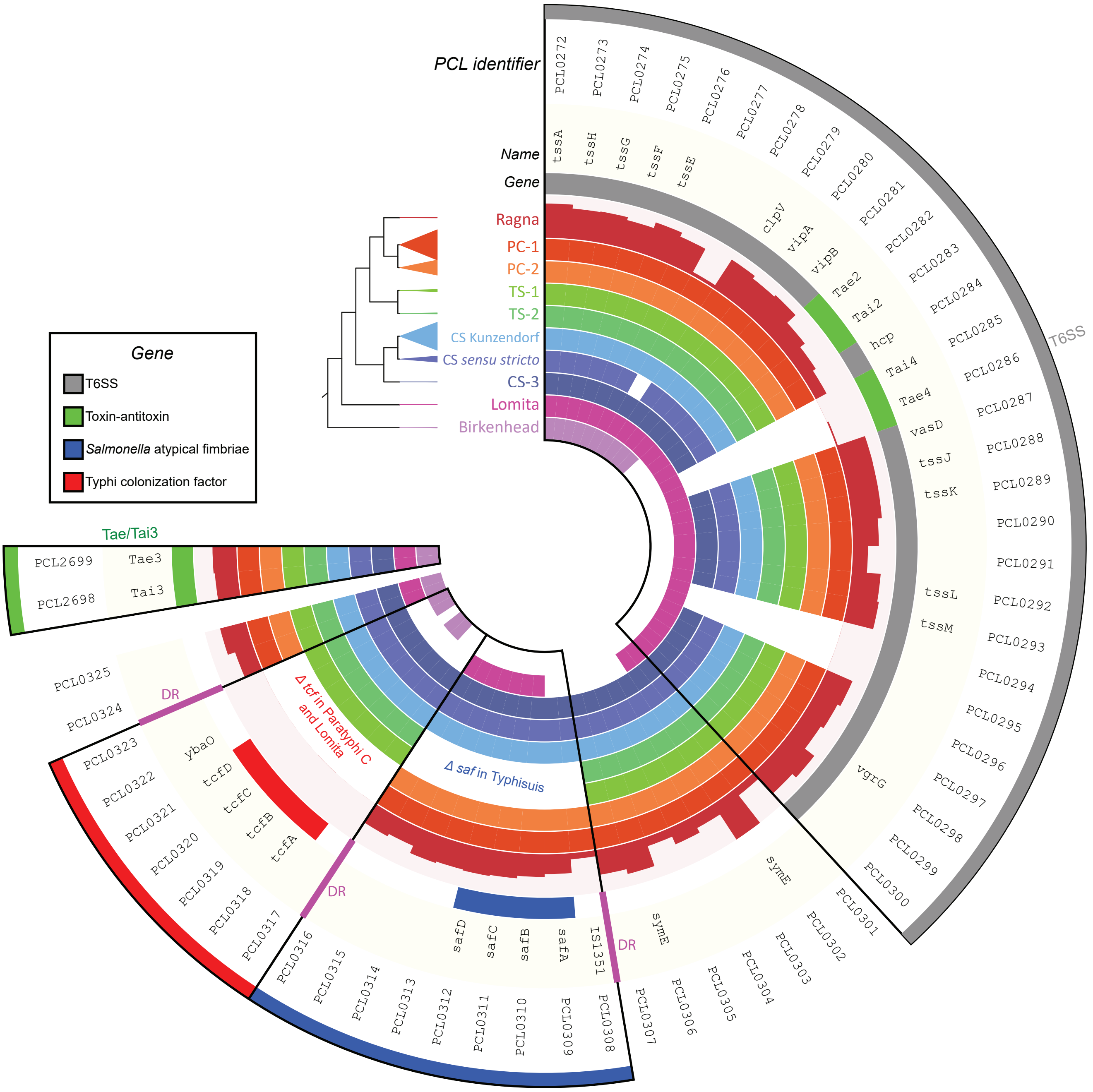

Figure S4. Variation of gene content of SPI-6 with sub-lineage. Related to Figure 3. SPI-6 may have been acquired by the Para C Lineage via horizontal gene transfer (HGT) after it split from Birkenhead because many T6SS genes within SPI-6 are absent in Birkenhead, including *tssL*, *tssM* and *vgrG* genes whereas all T6SS genes are present throughout the ParaC Lineage. The entire *tcf* (Typhi colonization factor) region has been deleted at direct-repeats (DR, pink) that are associated with IS1351 in Paratyphi C as well as Lomita. Similarly, the *saf* (*Salmonella* atypical fimbriae) cluster has been deleted at DR boundaries in Typhisuis. If SPI-6 were only imported once into the Para C Lineage, at least three independent deletions would be needed to account for these patterns. Alternatively, SPI-6 in Lomita may have been acquired by an independent HGT event. Two independent acquisitions could explain why the termini of the deletion of the *saf* genes in Lomita differed from the termini in Typhisuis, and why a Tae4/Tai4 toxin/antitoxin T6SS effector is found exclusively in Lomita's SPI-6. Furthermore, unlike other members of the Para C Lineage, the SNP density in Lomita's SPI-6 was fourfold higher (0.015) than in the core genome. An interactive version of this figure is available at Anvi'o server SPI-6.

Categories	Count
Lineages	
Choleraesuis var Kunzendorf	73
Choleraesuis *sensu stricto*	16
Choleraesuis CS-3	1
Paratyphi C PC-1	62
Paratyphi C PC-2	57
Ragna	1
Typhisuis TS-1	6
Typhisuis TS-2	3
Lomita	1
Collection years	
1200±50	1
1914-1954[1]	21
1955-1979	59
1980-1999	57
2000-2015	67
Continents	
Africa	65
Asia	18
Europe	73
North America	45
South America	2

Table S1. Numbers of genomes by lineage, year and continent from the Para C Lineage (Ragna plus 219 modern bacterial strains). Related to Figure 2.

[1]Strains M263 (NCTC20356) and M80 (NCTC348) from the Murray collection are listed under the category 1914-1954 because it has been documented [S1] that they were isolated before 1954. The year of isolation was completely unknown for 15 other bacterial strains, and the continental source was unknown for 17, including Lomita.

	No. of Islands	No. of genes	Total Length (bp)	Terminal nodes		Internal nodes	
				Gain	Loss	Gain	Loss
**Core genes**	N.A.	3,542	3,387,468	N.A.	N.A.	N.A.	N.A.
**Accessory genes**	227	3,123	2,436,825	63	130	37	81
**Prophages**							
Lambda supercluster	17	459	305,175	13	11	13	12
P2 supercluster	5	172	126,438	3	2	2	0
P1 supercluster	1	97	71,289	1	0	0	0
Mu supercluster	1	50	34,377	0	0	1	0
Others	8	21	15,364	2	3	0	3
**Subtotal:**	32	799	552,663	19	16	16	15
**ICE**	2	107	89,160	0	1	2	0
**IME**	16	86	65,079	7	3	4	4
**Plasmids**	37	1,377	1,073,457	31	11	10	3
**Other islands**	140	754	656,466	6	99	5	59

Table S2. Numbers of gene gain/loss events within the pan-genome of the Para C Lineage including Ragna (220 genomes). Related to Figure 3.

A list of all genomic islands is present in ParaC Pan-genome 7, and the branches on which these events occurred is illustrated in ParaC Pan-genome 1. The cumulative numbers of pseudogenes per branch can also be seen in Figure S3C. ParaC Pan-genome 4 provides a list of all genes and pseudogenes for each genome. N.A.: Not Applicable

Genes/GIs	Category	Description	Status	Citation
GI008	Pathogenicity island	SPI-6	Present in Para C Lineage.	
tcf	Host specificity / fimbriae	SPI-6 Typhi colonization factor	Deleted in Paratyphi C and Lomita	[S2,S3]
saf	fimbriae	SPI-6 *Salmonella* atypical fimbriae	Deleted in Typhisuis and Lomita	[S4]
GI107	Pathogenicity island	SPI-7	Present in all PC-1, PC-2 and Ragna. 4.5 kb internal deletion of 5 genes in PC-1.	Figure 2
tvi	Prevents Complement Receptor 3-mediated clearance	SPI-7 Vi capsular polysaccharide	All Paratyphi C	[S5,S6]
GI010A	prophage	P22-like (lambda)	Present in Ragna and one other Paratyphi C genome and all but four Choleraesuis genomes	Figure 2
GI025	prophage	ESSI-2-like (P2)	Present in Ragna and all but two Paratyphi C genomes	Figure 2 [S7]
GI029	Pathogenicity island	SPI-5	STM1089 disrupted; STM1092-STM1093 deleted in Choleraesuis	
GI081	prophage	SEN34 (Gifsy-2)	Absent in Typhisuis	Figure 2
pltAB	Host specificity	Typhi toxin	Absent	[S8]
gtgE	Host specificity	Rab32-Dependent Pathway to human restriction in Typhi	Pseudogene in Typhisuis	[S9]
sopD2 (SPI-2)	Host specificity	Rab32-Dependent Pathway to human restriction in Typhi	Present with SNP variation	[S9]
fimH	Host specificity / fimbriae	Host-specific adhesion	Deleted in Choleraesuis *sensu stricto*	[S10]
fepE	Serum resistance	LPS length regulation	Pseudogene in TS-2	[S11]
opvAB	Serum resistance	LPS length regulation	Present with SNP variation	[S12]
pgtE	Serum resistance	Cleaves complement factors B and H	SCP-P1 (GI1076) inserts upstream in some PC-1 strains and increases virulence in a murine model	[S13,S14]
rck	Serum resistance	Binds to complement factor H	Deleted in both Choleraesuis and modern Paratyphi C	[S15]
pagC	Serum resistance		Present with SNP variation	[S16]
cbi, cbo	Central metabolism	Vitamin B12 biosynthesis	Present with SNP variation	[S17,S18]
pdu	Central metabolism	Propanediol utilization	Deleted in Choleraesuis	[S17,S18]
eut	Central metabolism	Ethanolamine utilisation	Pseudogene in Choleraesuis and Typhisuis	[S17,S18]
ttr, phs, asr	Central metabolism	Tetrathionate reduction	Pseudogene in Choleraesuis *sensu stricto* and TS-2	[S17,S18]
bcs	Multicellular behaviour		Deleted in Choleraesuis var. Kunzendorf and disrupted in Typhisuis	[S19]
katE	Multicellular behaviour		Present with SNP variation	[S19]

Table S3. Distribution across the Para C lineage of selected GIs and genes associated with host specificity, serum resistance, adhesion, central metabolism and multicellular behaviour. Related to Figure 2.

Lineages	Subsamples: Paratyphi C		Subsamples: Para C Lineage w/o Lomita		Summary date estimate (BP)
	**Median date (BP)**	**Median CI95% (BP)**	**Median date (BP)**	**Median CI95% (BP)**	
PC-1	353	249 - 487	219	133 – 407	219-353 (133-487)
PC-2	556	390 - 788	330	191 – 556	330-556 (191-788)
Modern Paratyphi C (w/o Ragna)	664	456 - 922	456	251 – 816	456-664 (251-922)
Paratyphi C (including Ragna)	1526	1186 - 1975	1162	883 – 1766	1,162-1,526 (883-1,975)
Paratyphi C & Typhisuis	-	-	2663	1400 - 4683	2,663 (1,400-4,683)
Choleraesuis var. Kunzendorf	-	-	270	137 - 506	270 (137-506)
Choleraesuis *sensu stricto*	-	-	302	158 - 544	302 (158-544)
Choleraesuis	-	-	1811	755 - 3435	1,811 (755-3,435)
Paratyphi C, Typhisuis & Choleraesuis	-	-	3428	1707 - 6172	3,428 (1,707-6,172)

Table S4. Dating estimates of MRCAs within the Para C Lineage calculated by BEAST with 10 random subsamples of 50 genomes from Paratyphi C or from the Para C Lineage. Related to Figure 3.

Median date and Median CI95% refer to median values of median posterior dates and CI95% from non-recombinant core SNPs in 10 independent subsamples of 50 genomes including Ragna (see Date estimation 4) using either Paratyphi C or the Para C Lineage without Lomita (which lacked a date of collection).
Median clock rates used the following models and yielded the following molecular clock rates. Paratyphi C–Strict: $7.9x10^{-8}$ ($5.3x10^{-8}$-$1.1x10^{-7}$); Para C Lineage w/o Lomita– Relaxed: $1.5x10^{-7}$ ($6.9x10^{-8}$-$2.5x10^{-7}$).
Summary date estimates are according to estimates with the Para C Lineage, except for dates within Paratyphi C, in which case they represent the ranges of estimates from both subsamples.

Supplemental References

S1. Baker K.S., Burnett E., McGregor H., Deheer-Graham A., Boinett C., Langridge G.C., Wailan A.M., Cain A.K., Thomson N.R., Russell J.E. et al. (2015). The Murray collection of pre-antibiotic era *Enterobacteriacae*: a unique research resource**.** Genome Med. *7***:** 97.

S2. Folkesson A., Advani A., Sukupolvi S., Pfeifer J.D., Normark S., and Lofdahl S. (1999). Multiple insertions of fimbrial operons correlate with the evolution of *Salmonella* serovars responsible for human disease**.** Mol. Microbiol. *33***:** 612-622.

S3. Leclerc J.M., Quevillon E.L., Houde Y., Paranjape K., Dozois C.M., and Daigle F. (2016). Regulation and production of Tcf, a cable-like fimbriae from *Salmonella enterica* serovar Typhi**.** Microbiology *162***:** 777-788.

S4. Carnell S.C., Bowen A., Morgan E., Maskell D.J., Wallis T.S., and Stevens M.P. (2007). Role in virulence and protective efficacy in pigs of *Salmonella enterica* serovar Typhimurium secreted components identified by signature-tagged mutagenesis**.** Microbiology *153***:** 1940-1952.

S5. Wangdi T., Lee C.Y., Spees A.M., Yu C., Kingsbury D.D., Winter S.E., Hastey C.J., Wilson R.P., Heinrich V., and Baumler A.J. (2014). The Vi capsular polysaccharide enables *Salmonella enterica* serovar typhi to evade microbe-guided neutrophil chemotaxis**.** PLoS. Pathog. *10***:** e1004306.

S6. Wilson R.P., Winter S.E., Spees A.M., Winter M.G., Nishimori J.H., Sanchez J.F., Nuccio S.P., Crawford R.W., Tukel C., and Baumler A.J. (2011). The Vi capsular polysaccharide prevents complement receptor 3-mediated clearance of *Salmonella enterica* serotype Typhi**.** Infect. Immun. *79***:** 830-837.

S7. Lee Y.D., Chang H.I., and Park J.H. (2011). Complete genomic sequence of virulent *Cronobacter sakazakii* phage ESSI-2 isolated from swine feces**.** Arch. Virol. *156***:** 721-724.

S8. Galan J.E. (2016). Typhoid toxin provides a window into typhoid fever and the biology of *Salmonella* Typhi**.** Proc. Natl. Acad. Sci. U. S. A *113***:** 6338-6344.

S9. Spano S., Gao X., Hannemann S., Lara-Tejero M., and Galan J.E. (2016). A bacterial pathogen targets a host Rab-Family GTPase defense pathway with a GAP**.** Cell Host. Microbe *19***:** 216-226.

S10. Yue M., Han X., De M.L., Zhu C., Ma X., Zhang J., Wu R., Schmieder R., Kaushik R.S., Fraser G.P. et al. (2015). Allelic variation contributes to bacterial host specificity**.** Nat. Commun. *6***:** 8754.

S11. Crawford R.W., Wangdi T., Spees A.M., Xavier M.N., Tsolis R.M., and Baumler A.J. (2013). Loss of very-long O-antigen chains optimizes capsule-mediated immune evasion by *Salmonella enterica* serovar Typhi**.** MBio. *4*.

S12. Cota I., Sanchez-Romero M.A., Hernandez S.B., Pucciarelli M.G., Garcia-del P.F., and Casadesus J. (2015). Epigenetic Control of *Salmonella enterica* O-Antigen Chain Length: A Tradeoff between Virulence and Bacteriophage Resistance**.** PLoS. Genet. *11***:** e1005667.

S13. Ramu P., Tanskanen R., Holmberg M., Lahteenmaki K., Korhonen T.K., and Meri S. (2007). The surface protease PgtE of *Salmonella enterica* affects complement activity by proteolytically cleaving C3b, C4b and C5**.** FEBS Lett. *581***:** 1716-1720.

S14. Zou Q.H., Li Q.H., Zhu H.Y., Feng Y., Li Y.G., Johnston R.N., Liu G.R., and Liu S.L. (2010). SPC-P1: a pathogenicity-associated prophage of *Salmonella* paratyphi C**.** BMC. Genomics *11***:** 729.

S15. Ho D.K., Tissari J., Jarvinen H.M., Blom A.M., Meri S., and Jarva H. (2011). Functional recruitment of human complement inhibitor C4B-binding protein to outer membrane protein Rck of *Salmonella***.** PLoS. One. *6***:** e27546.

S16. Nishio M., Okada N., Miki T., Haneda T., and Danbara H. (2005). Identification of the outer-membrane protein PagC required for the serum resistance phenotype in *Salmonella enterica* serovar Choleraesuis**.** Microbiology *151***:** 863-873.

S17. Nuccio S.P. and Baumler A.J. (2014). Comparative analysis of *Salmonella* genomes identifies a metabolic network for escalating growth in the inflamed gut**.** MBio. *5***:** e00929-14.

S18. Langridge G.C., Fookes M., Connor T.R., Feltwell T., Feasey N., Parsons B.N., Seth-Smith H.M., Barquist L., Stedman A., Humphrey T. et al. (2015). Patterns of genome evolution that have accompanied host adaptation in *Salmonella***.** Proc. Natl. Acad. Sci. U. S. A *112***:** 863-868.

S19. Singletary L.A., Karlinsey J.E., Libby S.J., Mooney J.P., Lokken K.L., Tsolis R.M., Byndloss M.X., Hirao L.A., Gaulke C.A., Crawford R.W. et al. (2016). Loss of multicellular behavior in epidemic African nontyphoidal *Salmonella enterica* Serovar Typhimurium ST313 strain D23580**.** MBio. *7***:** e02265.
